# Acute effects of tissue flossing on ankle range of motion in athletes with limited ankle dorsiflexion: a randomized controlled trial

**DOI:** 10.1186/s13102-025-01484-w

**Published:** 2025-12-22

**Authors:** Hassan Daneshmandi, Mohammad Alghosi, Mohammad Alimoradi, Omid Monfaredian, Amirhossein Barati, Urs Granacher

**Affiliations:** 1 Department of Sports Injury and Corrective Exercise, Faculty of Physical Education and Sport Sciences, University of Guilan, Rasht, Iran; 2 Department of Physical Education, Technical and Vocational University (TVU), Tehran, Iran; 3 Department of Sports Injuries and Corrective Exercises, Faculty of Sports Sciences, Shahid Bahonar University of Kerman, Kerman, Iran; 4 HERC – Health, Exercise & Research Center, Mina Rashid, Dubai Maritime City, Dubai, United Arab Emirates; 5 Department of Sports Biomechanics, Faculty of Sports Sciences, Shahid Bahonar University of Kerman, Kerman, Iran; 6 Department of Sports Medicine and Health, Faculty of Physical Education, University of Shahid Behshti, Tehran, Iran; 7 Department of Sport and Sport Science, Exercise and Human Movement Science, University of Freiburg, Freiburg, Germany

**Keywords:** Mobility, Compression, Stretching, Athletic performance, Injury prevention

## Abstract

**Background:**

Restricted ankle dorsiflexion (ADF) is a common impairment in athletes, associated with decreased performance and increased risk of injury. Tissue flossing (TF) is an emerging technique proposed to rapidly improve joint range of motion (ROM), though its acute effects on athletes with limited ADF require further investigation. This study aimed to evaluate the acute effects of a single session of TF application on ADF and plantarflexion ROM in athletes with limited ADF.

**Methods:**

Forty-four male athletes aged 25.9 ± 4.4 years with ADF-ROM < 10° were randomly allocated to a TF intervention (*n* = 22) or a static stretching (SS) active control group (*n* = 22). The TF intervention used a standard figure-of-eight bandaging technique with 50–70% overlap. While wrapped on both legs, participants performed 3 sets of ankle pumps, squats, and lunges. The SS group performed 3 × 30 s stretches for the gastrocnemius and soleus muscles on both legs. Dorsiflexion and plantarflexion ROM were measured on the dominant and non-dominant leg at baseline, immediately post-intervention, and 1 h after post-tests (follow-up).

**Results:**

Group-by-time interactions showed significant effects on dorsiflexion ROM in both limbs (all *p* < 0.001; *d* = 0.37–0.43). Post-hoc tests indicated that TF had greater immediate effects on dorsiflexion ROM (dominant: *p* < 0.001; *d* = 1.57, non-dominant: *p* < 0.001; *d* = 1.52) than SS (dominant: *p* = 0.020; *d* = 0.53, non-dominant: *p* < 0.001; *d* = 0.33), with small but significant retention for TF at follow-up (dominant: *p* = 0.018; *d* = 0.25, non-dominant: *p* = 0.005; *d* = 0.11). A group-by-time interaction was also found for plantarflexion ROM in the dominant side (*p* < 0.001, *d* = 0.44). TF showed greater effects (*p* < 0.001; *d* = 0.62) than SS (*p* < 0.001; *d* = 0.30), with small retention gains (*p* < 0.001; *d* = 0.07).

**Conclusion:**

A single-session TF application in combination with physical exercise (e.g., squats) resulted in greater immediate dorsiflexion ROM improvements than traditional SS in athletes with ADF. However, due to the multimodal nature and longer duration of the flossing protocol, further research is needed to verify our results.

**Trial registration:**

This trial was registered at the Iranian Registry of Clinical Trials (Identifier: IRCT20230612058457N8) on August 29, 2025.

## Background

Restricted ankle dorsiflexion (ADF) is a frequent biomechanical limitation seen in athletes across a wide range of sports [1]. Adequate ADF is essential for effective movement and balance control, playing a key role in dynamic tasks such as sprinting, jumping, and quick changes of direction, which are influenced by neuromuscular control and postural stability factors associated with lower limb movement patterns [2, 3]. When ankle mobility is compromised, athletes often develop compensatory strategies that can decrease force production efficiency, hinder stability, and elevate the risk of injuries like ankle sprains, Achilles tendinopathy, and patellar tendinopathy [4–6]. These concerns are especially relevant during the performance of high-impact tasks such as squatting, cutting, and landing [4, 5, 7]. In this regard, female elite athletes demonstrated a higher prevalence of ankle instability compared to male athletes, with acrobatic athletes exhibiting a significantly greater prevalence than non-contact athletes [8]. Traditional interventions to improve ADF include static stretching (SS), soft tissue work, and manual therapy. However, these approaches typically require a long-term commitment and often yield inconsistent results [9–11]. Tissue flossing (TF) is an emerging technique designed to enhance the range of motion (ROM), alleviate pain, prevent injuries, and support an earlier return to competition for athletes. The method involves tightly wrapping a joint or muscle with a natural rubber floss band while performing passive twisting and active movements [12]. The approach gained popularity through the work of Starrett and Cordoza [13], who suggested that the benefits of TF are driven by fascial shearing and temporary restriction of blood flow to the muscle. Additionally, the combination of compression and movement during TF is believed to alter the interaction between the fascia and the neuromuscular system, enabling the fascia to stretch and move more freely. Previously, researchers have reported positive effects of TF on ankle ROM [2, 14, 15]. For example, a study conducted with recreational athletes found that a single 2-minute TF application significantly improved dorsiflexion ROM (10.9 ± 6.0 vs. 12.7 ± 6.5 cm ) compared to a control condition (11.4 ± 6.7 vs. 11.6 ± 6.5 cm) [16]. Despite growing interest in TF, the acute effects on ankle ROM in athletes with limited ADF have not been thoroughly investigated. Accordingly, it is timely to investigate the acute and follow-up effects of TF on ankle ROM in athletes. Establishing these effects is crucial, as acute mobility gains could directly enhance performance in tasks like squatting and jumping, and inform time-efficient warm-up strategies for injury prevention [2, 17]. Here, we aimed to evaluate the acute effects of TF on ADF and plantarflexion ROM in athletes with limited ADF. Based on the available literature [2, 15, 18], it was hypothesized that a single application of TF would produce significantly greater acute improvements in ADF and plantarflexion ROM compared with a single session of the SS program. It was further expected that these ROM gains would be retained 1 h after the post-tests [2].

## Methods

### Participants

This randomized controlled trial included 44 male athletes aged 18–35 years (mean age: 25.9 ± 4.4 years; height: 179.1 ± 3.3 cm; mass: 75.5 ± 3.1 kg) recruited from collegiate and semi-professional local sport teams in Shiraz, Iran, including soccer, basketball, badminton, and tennis. According to the participant classification framework by McKay et al. [19], this cohort is representative of Tier 3 athletes, indicating they were highly trained individuals competing in structured leagues and engaging in regular, supervised training for more than 8 h per week. An a priori power analysis was calculated based on findings from a related study on the effects of TF on dorsiflexion ROM in athletes with chronic ankle instability [20]. As input parameters, a Cohen’s f effect size of f = 0.28 was used for the primary outcome, dorsiflexion ROM. In addition, an alpha error of *p* < 0.01 was included (5% probability of type I error), and β = 0.90 (90% statistical power). The power analysis revealed that at least 40 participants would be required to achieve significant and medium-sized group-by-time interactions. To account for potential dropouts, a target sample size of 44 (10%) was initially set.

**Fig. 1:**
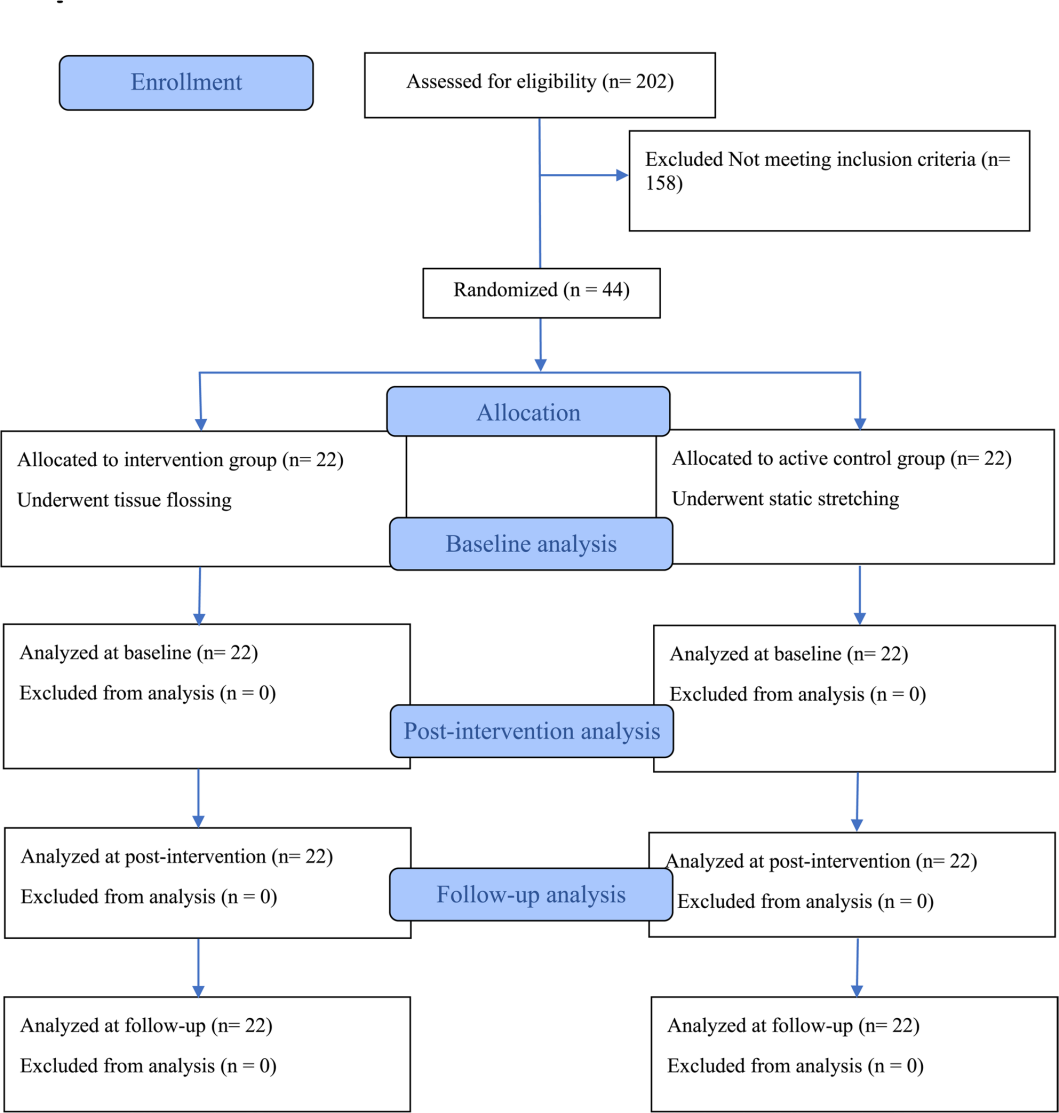
CONSORT 2025 flow diagram of participant enrollment, allocation, and analysis

Inclusion criteria were as follows: (a) dorsiflexion ROM < 10º, assessed via the weight-bearing lunge test using a goniometer (as detailed in the Measurements section), and (b) no history of fracture, surgery, or moderate-to-severe soft tissue injury in the past six months. Exclusion criteria were as follows: (a) use of an ankle brace during the study, (b) unwillingness to continue participation at any point, and (c) use of performance-enhancing therapies or supplements that could affect mobility or strength. Prior to data collection, all subjects provided written informed consent. Additionally, the participants completed an athlete injury questionnaire. The ethical approval for this study was provided by the Institutional Review Board of Guilan University (IR.GUILAN.REC.1404.017) on April 14, 2025. Written informed consent was obtained from all participants prior to their participation in the study. Subsequently, participants were randomly allocated into the experimental groups by one of the researchers in a 1:1 ratio using the www.randomizer.orgwebsite. The allocation resulted in an intervention group receiving TF (*n* = 22) and an active control group performing SS (*n* = 22). The study was registered with a clinical trial registry (IRCT20230612058457N8) on August 29, 2025. Additionally, the authors followed the principles of the Declaration of Helsinki. This study adheres to the CONSORT (Consolidated Standards of Reporting Trials) guidelines for the reporting of randomized controlled trials [21]. Fig. 1 presents the study progression, detailing participant enrollment, allocation, and analysis.

### Procedure

All participants attended a familiarization session 48 h before baseline testing to learn the assessment protocols and minimize learning effects. During this session and the baseline assessment, a subsample of 15 participants (from both TF and SS groups) completed repeated measurements to evaluate test–retest reliability. Intraclass correlation coefficients (ICCs) were calculated for all outcome variables, with ICC > 0.80 considered indicative of good reliability. Standard error of measurement (SEM), minimal detectable change (MDC_95%_) and coefficient of variation (CV%) values were also computed (Table 1).

**Table 1:** Reliability and measurement error metrics

Variable	ICC (1, 2)	SEM	MDC_95%_	CV%
ADF-ROM (°) Dominant leg	0.92 [0.78–0.97]	0.27	0.75	19.74
ADF-ROM (°) Non-dominant leg	0.93 [0.79–0.98]	0.22	0.61	14.34
APF-ROM (°) Dominant leg	0.97 [0.81–0.99]	0.43	1.18	5.67
APF-ROM (°) Non-dominant leg	0.98 [0.95–0.99]	0.39	1.08	4.93

*Abbreviations*: *ICC* Intra-class coefficient, *SEM* Standard error mean, *MDC* Minimal detectable change, *CV* Coefficient of variation, *ADF* Ankle dorsiflexion, *ROM* Range of motion, *APF* Ankle plantarflexion

**Fig. 2:**
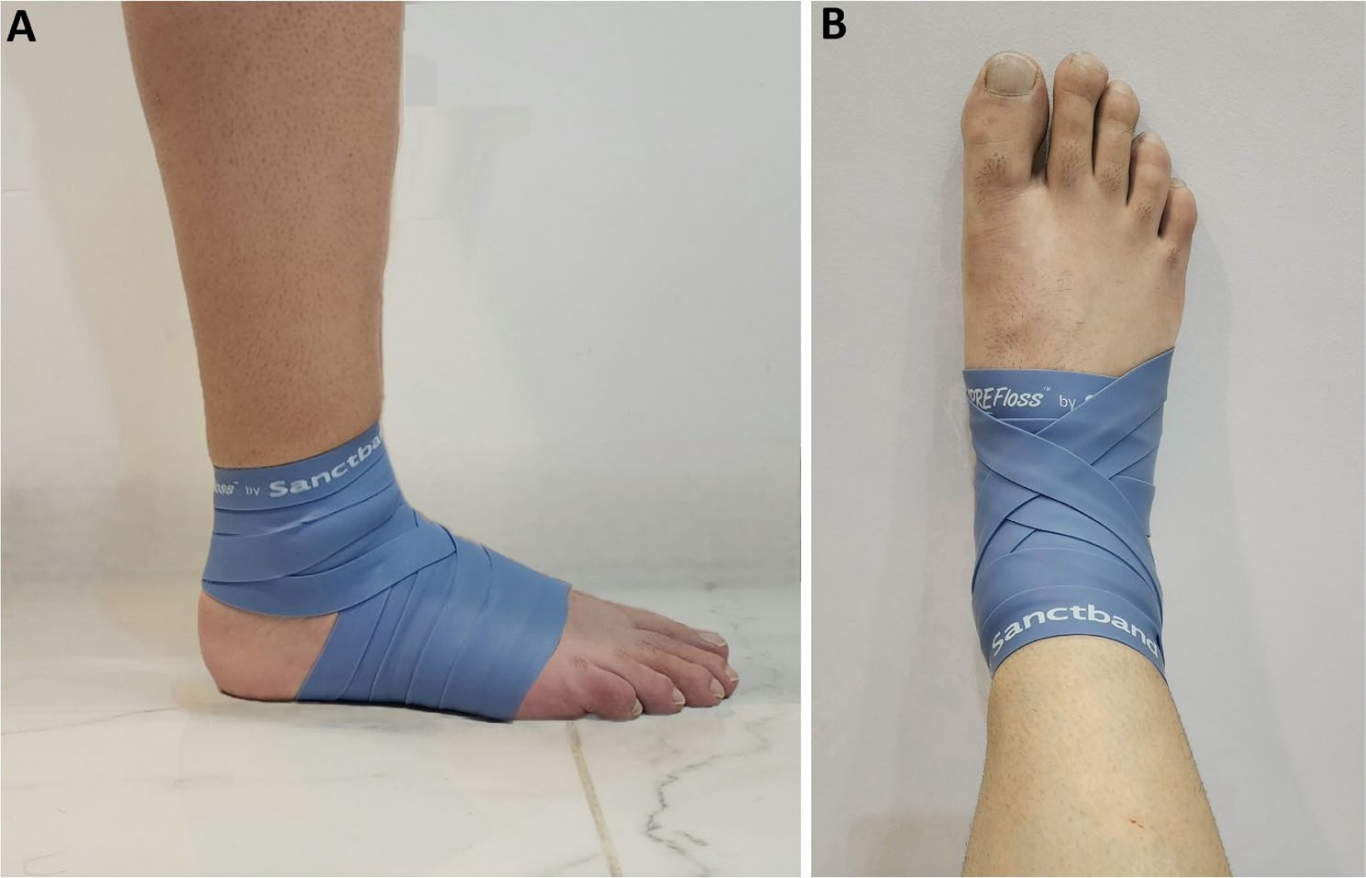
The floss band ankle bandaging technique [2, 23]. **A** Lateral view; (**B**) Anterior view

**Fig. 3:**
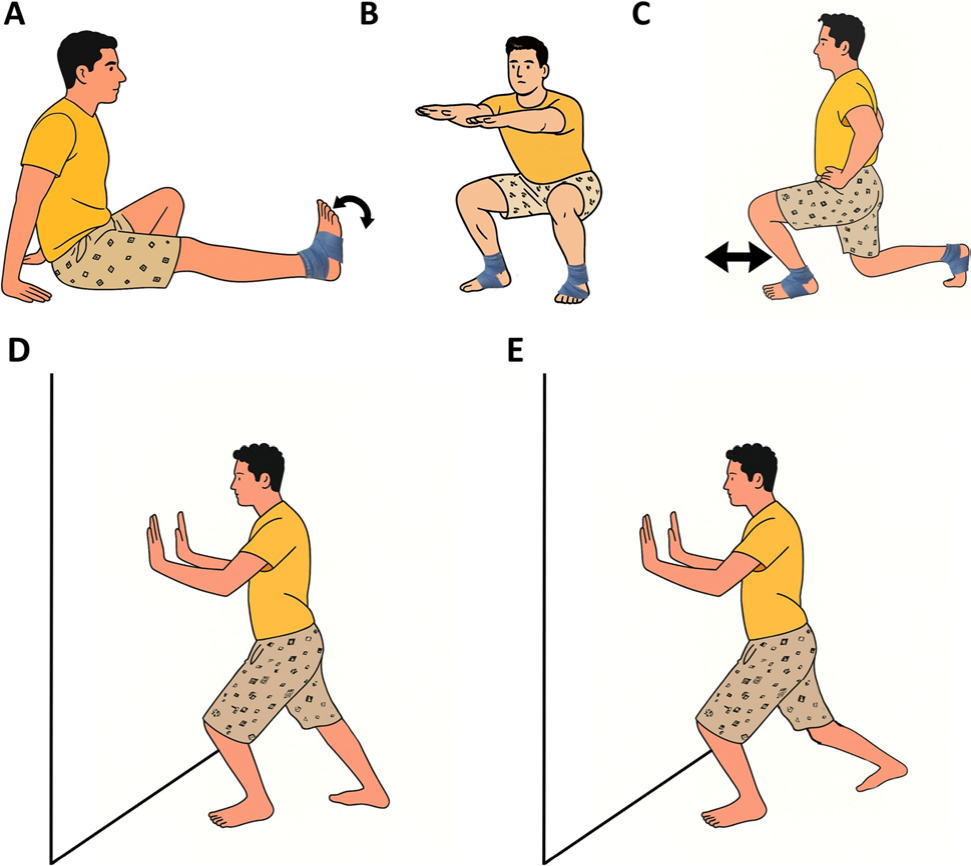
Description of exercises according to the experimental groups: tissue flossing with ankle pumps, squats, lunges (**A**, **B**, and **C**) and static stretching (**D **and **E**)

On the testing day, participants first completed a standardized five-minute cycling warm-up. They were then assigned to either the TF group or the SS group. In the TF group, an elastic floss band (Sanctband COMPRE Floss, Blueberry; PENTEL SDN.BHD., Shah Alam, Malaysia), sized 2 m × 5 cm, was wrapped around the ankle joint using a standard technique. This involved two circular wraps around the foot’s transverse arch, followed by three figure-of-eight wraps (from lateral malleolus, around the Achilles tendon, to medial malleolus, and back), with each subsequent wrap overlapping the previous by approximately 50–70% (Fig. 2) [2, 22]. Moreover, interface pressure between the skin and the floss band was measured to assess the level of compression (in mmHg) achieved by the wrapping technique. A Kikuhime pressure monitor sensor (MediGroup, Melbourne, Australia) was placed on the anterior aspect of the tibia at the midline between the lateral and medial malleolus during wrapping to ensure consistent and safe pressure application. This device has an ICC of 0.99, indicating excellent reliability for use in sports medicine settings [23]. While the band was applied, participants performed ankle pumps, bodyweight squats, and lunges in three sets according to the protocol (Fig. 3A and B, and 3C). In the SS group, participants performed SS of the gastrocnemius and soleus muscles (Fig. 3D and E). Each stretch was held for 30 s and repeated three times, with 15 s of rest between sets. Testing was performed at three time points: baseline (before the intervention), immediately post-intervention (5 min after completion), and follow-up (1 h after completion). At each time point, participants completed the following tests in randomized order: (1) dorsiflexion ROM, and (2) plantarflexion ROM. A two-minute rest was provided between trials. All measurements were performed on both the dominant and nondominant legs, with dominance defined as the preferred kicking leg [24]. Moreover, no adverse events occurred in either of the studied groups. Table 2 outlines the characteristics of the intervention protocols.

**Table 2:** Characteristics of the intervention programs

Interventions	
Tissue flossing	Static stretching
• **Ankle pumps** o Sets: 3 o Duration: 60 s/set o Concentric: 1 s (plantarflexion) o Eccentric: 2 s (dorsiflexion) o Rest: 1 s at end ranges	• **M. gastrocnemius** o Stretch exercise: Standing Wall Stretch (straight back leg, knee extended) o Sets: 3 o Duration: 30 s per set o Rest: 15 s between sets
• **Squats with the own body mass** o Sets: 3 o Duration: 30 s/set o Concentric: 1–2 s o Eccentric: 3–4 s o Rest: 1 s at bottom	• **M. soleus** o Stretch exercise: Standing Wall Stretch (back leg bent, knee slightly flexed) o Sets: 3 o Duration: 30 s per set o Rest: 15 s between sets
• **Weight-bearing lunges** o Sets: 3 o Duration: 45 s/set o Concentric: 2 s o Eccentric: 3 s o Rest: 2 s at end ROM	

*Abbreviations*: *S* Second, *ROM* Range of motion, *M* Muscle

### Measurements

#### Dorsiflexion ROM

**Fig. 4:**
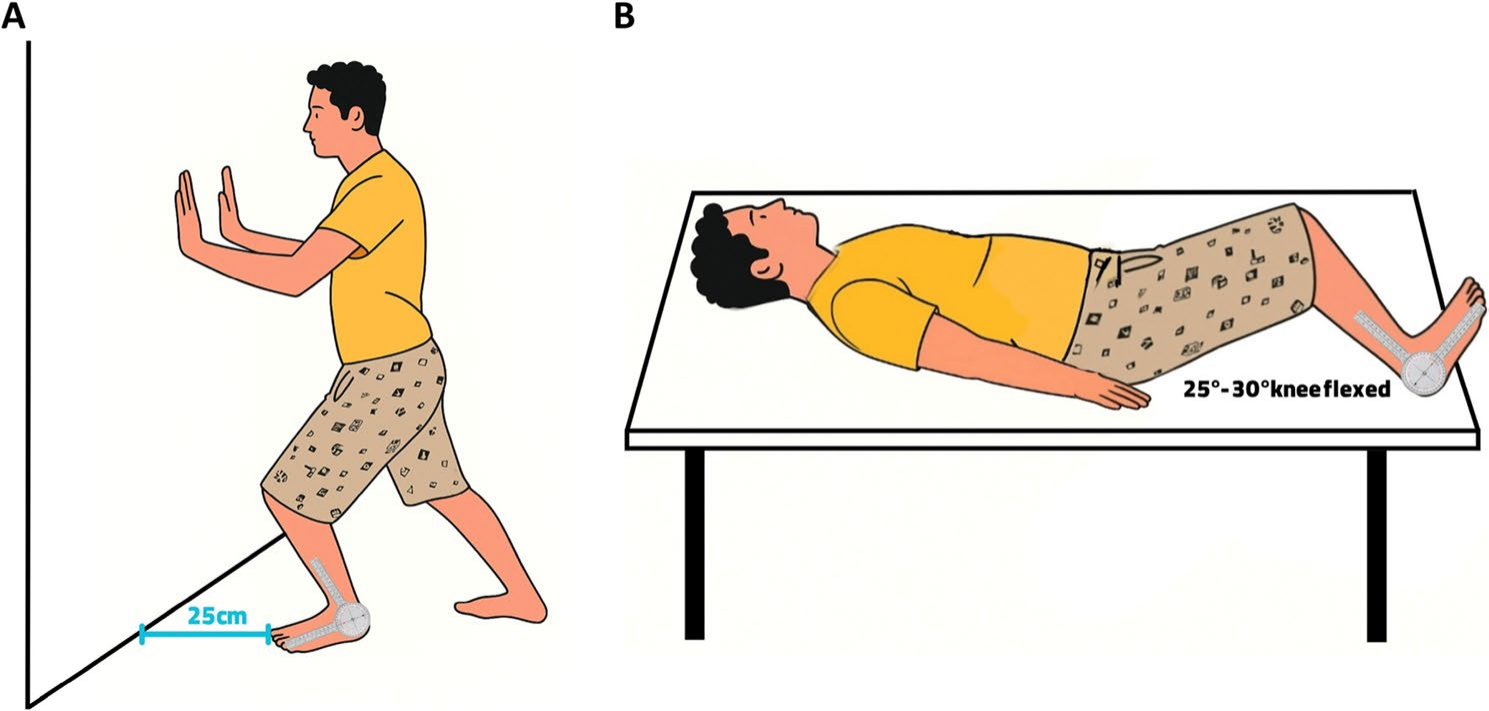
Ankle range of motion testing. **A** Dorsiflexion measurement; **(B)** Plantarflexion measurement

ADF was assessed with the weight-bearing lunge test. Participants stood barefoot, facing a wall, with their great toe positioned 25 cm from the wall. With the test limb forward, the knee was kept in line with the second toe, and the heel remained in contact with the floor. The participant advanced toward the wall in 1 cm increments until the knee touched the wall without lifting the heel. The goniometer axis was centered over the lateral malleolus; the stationary arm was aligned with the fibula, and the moving arm with the fifth metatarsal. The dorsiflexion angle at the final position was recorded [25] (Fig. 4A).

#### Plantarflexion ROM

Participants were evaluated barefoot, supine on the bed with the knees flexed 25–30°. The feet were positioned in the neutral position (90°). The goniometer axis was placed over the lateral malleolus, with the stationary arm aligned parallel to the lateral aspect of the fibula and the moving arm aligned parallel to the lateral surface of the fifth metatarsal [26] (Fig. 4B).

### Statistics

All data were analyzed using SPSS software (version 27.0; IBM Corp., Armonk, NY, USA). The Shapiro-Wilk test was used to assess the normality of the data, with *p* > 0.05 indicating a normal distribution. To evaluate the main time and group effects, as well as their interactions (group-by-time), a repeated measures 2 × 3 ANOVA was computed for all dependent variables, with the factors being group (TF, SS) and time (baseline, post-intervention, follow-up). When a significant interaction was identified, post hoc pairwise comparisons were performed using t-tests with the Bonferroni correction. Effect sizes were reported for all significant findings to quantify their practical relevance. For the main effects and interactions derived from the repeated-measures ANOVA, partial eta squared (η²) was calculated, converted into Cohen’s *d*. Within-group Cohen’s *d* effect sizes were classified as small (0.00 < *d* < 0.49), moderate (0.50 ≤ *d* < 0.80), and large(*d* > 0.80) [27]. The significance level was set at *p* < 0.05, and the confidence interval was 95% for all analyses.

## Results

**Fig. 5:**
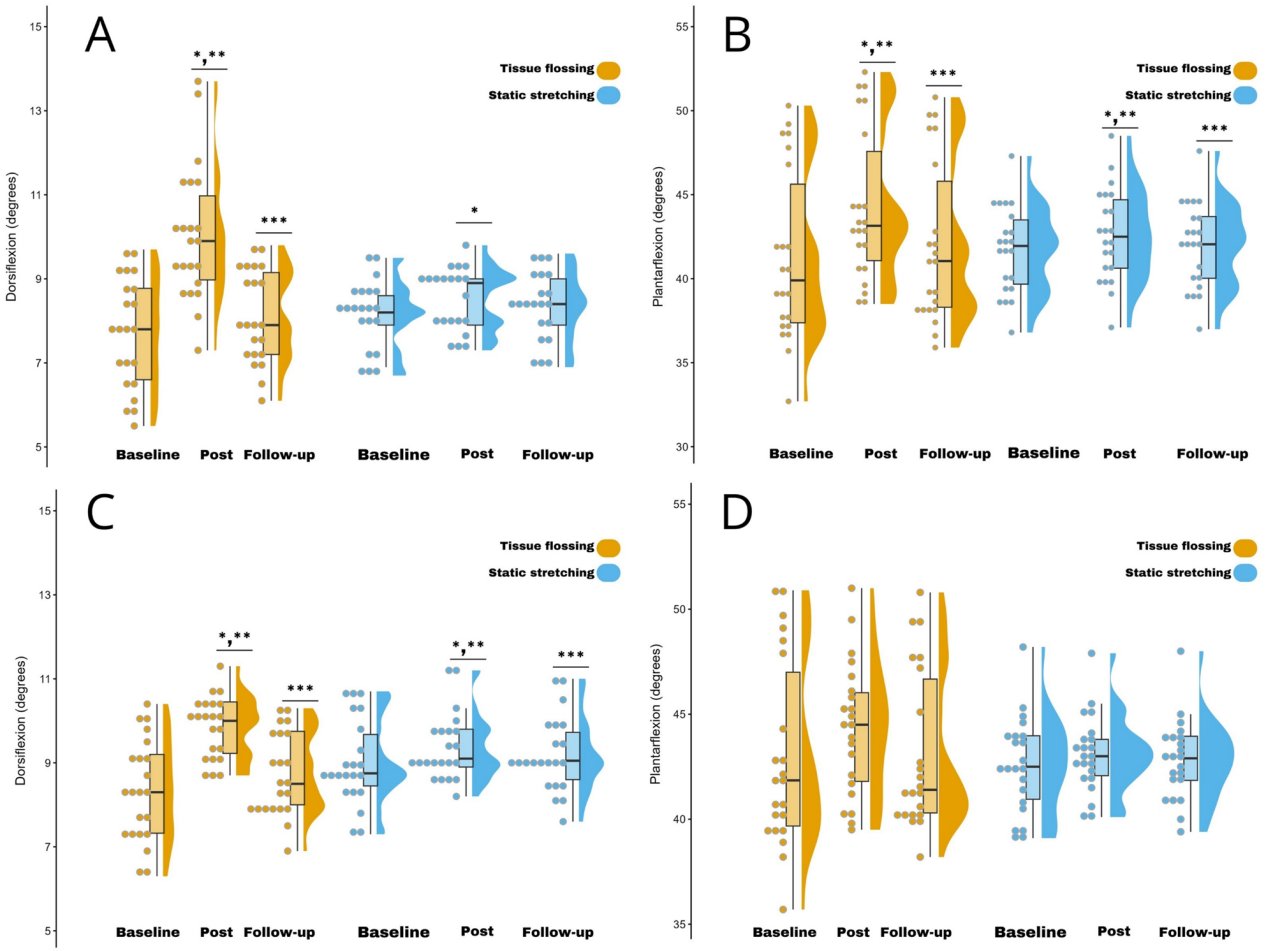
Changes in ankle dorsiflexion and plantarflexion range of motions across baseline, post-intervention, and follow-up assessments. **A** Dorsiflexion of the dominant leg; (**B**) Plantarflexion of the dominant leg; (**C**) Dorsiflexion of the non-dominant leg; (**D**) Plantarflexion of the non-dominant leg.* Significant difference compared to baseline (*p* < 0.05); ** Significant difference compared to follow-up (*p* < 0.05); *** Significant difference compared to baseline (*p* < 0.05)

All variables displayed a normal distribution (*p* > 0.05). Changes in the dependent variables over time for both groups are presented in Table 3 and depicted in Fig. 5. Statistical analysis revealed a significant, moderate main effect of time (F = 45.23, *p* < 0.001, *d* = 0.51) and a significant, small group-by-time interaction (F = 25.54, *p* < 0.001, *d* = 0.37), while no significant main effect of group was observed (F = 0.87, *p* = 0.35, *d* = 0.02) for dominant leg dorsiflexion ROM. Post-hoc comparisons indicated that within the TF group, post-intervention values were significantly greater than both baseline ( *p* < 0.001, *d* = 1.57, Δ29.87%, 95% CI 1.70–2.95) and follow-up ( *p* < 0.001, *d* = 1.47, Δ25%, 95% CI 1.47–2.40), each with a large magnitude. Additionally, follow-up values remained significantly higher than baseline, albeit with a small magnitude (*p* = 0.018, *d* = 0.25, Δ3.89%, 95% CI 0.07–0.69). Moreover, the SS group showed a significant, moderate improvement from baseline to post-intervention (*p* = 0.020, *d* = 0.53, Δ4.93%, 95% CI 0.06–0.69). For the non-dominant leg, results showed a significant moderate main effect of time (F = 80.71, *p* < 0.001, *d* = 0.65) and a significant small group-by-time interaction (F = 32.28, *p* < 0.001, *d* = 0.43), but no significant group effect (F = 0.60, *p* = 0.44, *d* = 0.01). For the TF group, post-hoc analysis revealed that post-intervention values were significantly higher than both baseline ( *p* < 0.001, *d* = 1.52, Δ18.07%, 95% CI 1.15 to 1.85) and follow-up (*p* < 0.001, *d* = 1.36, Δ12.64%, 95% CI 0.82 to 1.36), with large magnitudes, and that follow-up values were also significantly higher than baseline with a small magnitude ( *p* < 0.001, *d* = 0.37, Δ4.81%, 95% CI 0.22 to 0.59). For the SS group, post-intervention scores were significantly higher than both baseline (*p* < 0.001, *d* = 0.33, Δ3.33%, 95% CI 0.17 to 0.55) and follow-up (*p* = 0.002, *d* = 0.25, Δ2.19%, 95% CI 0.07 to 0.28) with small effect sizes. Furthermore, follow-up scores remained significantly higher than baseline scores, also with a small magnitude (*p* = 0.005, *d* = 0.11, Δ1.11%, 95% CI 0.06 to 0.30).

**Table 3:** Descriptive statistics for ankle dorsiflexion and plantarflexion ROM (°) across testing sessions in the studied groups

Outcome	Group	Baseline Mean ± SD	Post-intervention Mean ± SD	Follow-up Mean ± SD
Dorsiflexion-DL	TF	7.7 ± 1.3	10.0 ± 1.6	8.0 ± 1.0
	SS	8.1 ± 0.8	8.5 ± 0.7	8.3 ± 0.7
Dorsiflexion-NDL	TF	8.3 ± 1.2	9.8 ± 0.7	8.7 ± 0.9
	SS	9.0 ± 1.0	9.3 ± 0.8	9.1 ± 0.8
Plantarflexion-DL	TF	41.2 ± 5.1	44.2 ± 4.4	42.0 ± 4.8
	SS	41.7 ± 2.5	42.5 ± 2.7	41.9 ± 2.5
Plantarflexion-NDL	TF	42.9 ± 4.4	44.2 ± 3.1	43.1 ± 3.7
	SS	42.5 ± 2.2	43.0 ± 1.8	42.8 ± 1.8

Abbreviations: *ROM* Range of motion, *DL* Dominant leg, *NDL* Non-dominant leg, *TF* Tissue flossing, *SS* Static stretching, *SD* Standard deviation

For dominant leg plantarflexion ROM, a significant moderate main effect of time (F = 110.46, *p* < 0.001, *d* = 0.72) and a significant small group-by-time interaction (F = 33.56, *p* < 0.001, *d* = 0.44) were observed. No significant main effect of group was found (F = 0.13, *p* = 0.71, *d* = 0.003). Post-hoc analysis for the TF group showed that post-intervention values were significantly higher than both baseline and follow-up values. The improvement from baseline was of moderate magnitude (*p* < 0.001, *d* = 0.62, Δ7.28%, 95% CI 2.42–3.68), while the improvement over follow-up was of small magnitude (*p* < 0.001, *d* = 0.47, Δ5.23%, 95% CI 1.70–2.73). Furthermore, follow-up values remained significantly higher than baseline with a small magnitude (*p* < 0.001, *d* = 0.16, Δ1.94%, 95% CI 0.47–1.19). Similarly, the SS group showed significantly greater post-intervention scores than both baseline (*p* < 0.001, *d* = 0.30, Δ 1.91%, 95% CI 0.57–1.20) and follow-up ( *p* < 0.001, *d* = 0.23, Δ1.43%, 95% CI 0.34–0.90), with small magnitudes; follow-up scores also significantly exceeded baseline with small magnitude (*p* < 0.001, *d* = 0.07, Δ0.47%, 95% CI 0.17–0.34). In the non-dominant leg, a significant but small main effect of time was found (F = 3.72, *p* = 0.029, *d* = 0.09), while neither the group-by-time interaction (F = 1.01, *p* = 0.36, *d* = 0.02) nor the group effect (F = 0.59, *p* = 0.44, *d* = 0.01) were significant.

## Discussion

The primary aim of this study was to investigate the acute effects of a single TF intervention versus SS on ADF and ROM in athletes with limited ADF. The key finding was that TF elicited significantly greater improvements in dorsiflexion ROM in both the dominant and non-dominant limbs compared to SS. Furthermore, a small portion of this improvement was retained at the 1-hour follow-up. Furthermore, for plantarflexion ROM in the dominant limb, the TF intervention elicited significantly greater immediate improvements than those achieved through SS. Taken together, these findings suggest that a package combining TF with exercises yields greater immediate ROM improvements than a traditional static-stretching routine in this study. This is an important distinction for athletic populations where time-efficient methods for immediate performance preparation are highly valued.

The significant and substantial immediate increases in dorsiflexion ROM following TF (*d* = 1.57 for the dominant limb, *d* = 1.52 for the non-dominant limb) align with the findings of previous studies. A previous study reported that flossing of the ankle joint increased acute dorsiflexion ROM [2]. Furthermore, another study reported that flossing of the gastrocnemius muscle increased acute dorsiflexion ROM [28]. By enhancing ankle mobility, these improvements could potentially mitigate the risk of severe injuries, including anterior cruciate ligament injuries, which are prevalent in athletic populations [1, 29, 30]. While band flossing may enhance ROM, the precise physiological mechanisms responsible for these effects are not well-defined. Since the current study did not assess potential mechanisms, any discussion of them is necessarily theoretical. Prior research has theorized that the improvements in muscle flexibility it provides could stem from either mechanical changes in the tissue or sensory adaptations in the nervous system, such as an increased pain threshold [31]. An additional proposal suggests that flossing may work by causing fascial shearing and temporarily restricting blood flow to the muscle [13]. Fascia is a multi-layered system of fibrous connective tissue that permeates and surrounds muscles, joints, nerves, and blood vessels [32, 33]. Healthy function relies on the smooth gliding of these layers against each other during movement [34]. A key property of fascia is thixotropy: its ability to change from a gel-like solid to a more fluid solution when heat or mechanical pressure (like that from flossing) is applied. This reduces its viscosity and elasticity, potentially enhancing movement [35]. The application of a floss band generates significant mechanical compression around a muscle, which retains heat produced by elevated intramuscular pressure and subsequent contractions. It is hypothesized that this thermal and mechanical energy decreases the viscoelastic properties of the fascia, ultimately allowing for improved muscle elongation [36]. In addition to these fascial and mechanical explanations, it is important to consider the role of sensory adaptations. Evidence indicates that the acute ROM gains following flossing are most consistent with increases in stretch tolerance or pain threshold rather than reductions in passive stiffness [12]. Circumferential pressure applied by the floss band stretches the skin and underlying tissue, which can stimulate cutaneous and muscle mechanoreceptors, thereby modulating sensory feedback and altering the perception of stretch or discomfort [12]. This mechanism provides a plausible explanation for the large immediate increases in dorsiflexion observed in the present study. Moreover, the TF intervention also elicited a significant immediate improvement in plantarflexion ROM specifically in the dominant limb (*d* = 0.62). This finding extends beyond the expected improvements in dorsiflexion, suggesting that the benefits of flossing may not be limited to the antagonistic musculature but could also enhance the function of the agonist muscles. While SS is known to potentially inhibit maximal force production [37], the combination of compression and exercises during flossing may act as a form of neuromuscular priming for the plantarflexors. The compression could facilitate muscle spindle activity and enhance neural drive, leading to a greater voluntary ROM [12]. The fact that this effect was observed only in the dominant leg warrants further investigation but may be related to limb-specific neural adaptations and greater motor unit recruitment capabilities in the preferred limb. A further distinction between TF and traditional stretching lies in the contraction of the target muscle under compression. Unlike SS, which emphasizes antagonist activity, TF requires repeated activation of the compressed muscle itself [12]. We emphasize that these mechanistic interpretations remain speculative in the absence of a movement-matched, no-band comparator or a sham-band control.

From a practical standpoint, TF may represent a valuable addition to warm-up routines for athletes with restricted ankle mobility. The technique produces rapid improvements in ROM, which may translate to more efficient movement patterns during high-demand activities such as squatting, cutting, or landing. Given the time constraints of training and competition, these immediate effects are particularly relevant to coaches, clinicians, and athletes seeking quick mobility gains. At the same time, because our TF protocol combined compression with dynamic, full-ROM tasks and involved a longer activity bout than the static-stretching routine, the package effect likely reflects both the nature and the duration of the exposure.

Several limitations must be acknowledged. The intervention arms differed in content: TF combined compression with ankle pumps, squats, and lunges, whereas the comparison arm involved only static stretching exercises. Thus, we cannot determine whether similar benefits would arise from the same exercise protocol performed without the band. Future trials should therefore include an exercise/no-band group and a sham-band condition to isolate the effects of compression. The total exposure time also differed between conditions (i.e., 6.75 min for the exercise protocol including squats versus 3 min of SS). Extended, task-rich exposure may partly explain larger immediate ROM gains; studies matching session duration and repetition, or using a 2 × 2 design (band × movement), are needed to explore dose–response relationships. We only assessed joint ROM and did not include physiological or neuromechanical measures, so we cannot determine if gains reflect tissue property changes or increased stretch tolerance. Future studies should include these measures to clarify mechanisms. Participants and outcome assessors were not blinded to the condition, so placebo or expectancy effects cannot be ruled out. Incorporating a sham-flossing condition (minimal compression) and blinding assessors would help distinguish between physiological and perceptual influences. The sample consisted solely of young male athletes, which limits generalizability to females and older populations. Only short-term effects were assessed; therefore, long-term adaptations remain unknown. Finally, functional outcomes (e.g., jump performance or injury rates) were not evaluated.

## Conclusion

In summary, in athletes with limited ADF, a brief protocol combining TF with physical exercise (e.g., squats) yielded greater immediate increases in dorsiflexion (and, in the dominant limb, plantarflexion) ROM than a short static-stretching routine; however, given the multimodal content and longer duration of the TF condition, causal attribution to compression alone is not yet justified.

## Data Availability

The data that supports the findings of this study are available in the Zenodo data repository at https://doi.org/10.5281/zenodo.17051703.

## Abbreviations

ADF: Ankle Dorsiflexion
TF: Tissue Flossing
ROM: Range of Motion
SS: Static Stretching
ICC: Intra-Class Coefficient
SEM: Standard Error of Measurement
MDC: Minimal Detectable Change
CV: Coefficient of Variation
CONSORT: Consolidated Standards of Reporting Trials
